# Pepper Plants Harboring *L* Resistance Alleles Showed Tolerance toward Manifestations of Tomato Brown Rugose Fruit Virus Disease

**DOI:** 10.3390/plants11182378

**Published:** 2022-09-12

**Authors:** Or Eldan, Arie Ofir, Neta Luria, Chen Klap, Oded Lachman, Elena Bakelman, Eduard Belausov, Elisheva Smith, Aviv Dombrovsky

**Affiliations:** 1 Department of Plant Pathology and Weed Research, ARO, The Volcani Center, Rishon LeZion 7505101, Israel; 2 The Robert H. Smith Faculty of Agriculture, Food and Environment, The Hebrew University of Jerusalem, Rehovot 761001, Israel; 3 Department of Ornamental Plants and Agricultural Biotechnology, Agricultural Research Organization, The Volcani Center, 68 HaMaccabim Road, P.O. Box 15159, Rishon LeZion 7505101, Israel

**Keywords:** transient ToBRFV systemic infections, *L* resistance alleles, ToBRFV-infected *L*^0^ pepper seeds, root inoculation, foliar inoculation

## Abstract

The tobamovirus tomato brown rugose fruit virus (ToBRFV) infects tomato plants harboring the *Tm-2*^2^ resistance allele, which corresponds with tobamoviruses’ avirulence (*Avr*) gene encoding the movement protein to activate a resistance-associated hypersensitive response (HR). ToBRFV has caused severe damage to tomato crops worldwide. Unlike tomato plants, pepper plants harboring the *L* resistance alleles, which correspond with the tobamovirus *Avr* gene encoding the coat protein, have shown HR manifestations upon ToBRFV infection. We have found that ToBRFV inoculation of a wide range of undefined pepper plant varieties could cause a “hypersensitive-like cell death” response, which was associated with ToBRFV transient systemic infection dissociated from disease symptom manifestations on fruits. Susceptibility of pepper plants harboring *L*^1^, *L*^3^, or *L*^4^ resistance alleles to ToBRFV infection following HRs was similarly transient and dissociated from disease symptom manifestations on fruits. Interestingly, ToBRFV stable infection of a pepper cultivar not harboring the *L* gene was also not associated with disease symptoms on fruits, although ToBRFV was localized in the seed epidermis, parenchyma, and endothelium, which borders the endosperm, indicating that a stable infection of maternal origin of these tissues occurred. Pepper plants with systemic ToBRFV infection could constitute an inoculum source for adjacently grown tomato plants.

## 1. Introduction

In plant–virus interactions, a hypersensitive response (HR) is a manifestation of localized cell death associated with a resistance gene response toward the pathogen. However, manifestations of localized cell death response are not always associated with resistance toward pathogen systemic infections. Whereas HR is a characteristic of a resistance gene defense response, other factors cause a “hypersensitive-like cell death” response, which has been associated with plant susceptibility to viral systemic infections [1,2,3]. In addition, it has been shown that at temperatures above 30 °C, even HR appearances in plants harboring resistance genes were not of the characteristic resistance-associated form with defined boundaries. HRs formed at high temperatures had diffused boundaries and were associated with plant susceptibility to tobamovirus systemic infections [4,5].

*Tm-2*^2^ in tomatoes is an allele of a nucleotide-binding leucine-rich repeat (NB-LRR) encoding gene of durable resistance. In the presence of the avirulence (*Avr*) gene encoding the movement protein (MP) of tomato mosaic virus (ToMV) and tomato mottle mosaic virus (ToMMV), HRs are manifested [6,7]. The tobamovirus tomato brown rugose fruit virus (ToBRFV) infects tomato plants [8] and overcomes the *Tm-2*^2^ resistance allele [9]. ToBRFV has caused severe losses to tomato crops worldwide [10]. ToBRFV is a single-stranded positive-sense RNA virus (+ssRNA). The viral genome encodes six proteins: a 126 kDa silencing suppressor; a 186 kDa replicase complex, which is a read-through translation of the 126 kDa encoding region; a ~30 kDa MP; a ~17 kDa coat protein (CP); and two putative proteins of ~54 kDa and 4–5 kDa. The 126 kDa silencing suppressor of tobamoviruses interferes with the regulation of small RNAs, including small interfering RNAs (siRNAs) and cellular microRNAs (miRNAs) [11].

Recently, there have been several reports on the possible association between ToBRFV infection of pepper plants and severe disease symptoms on leaves and fruits that were sometimes apparent at high environmental temperatures [12,13,14,15]. Discolored leaves and rugose fruits were reported in pepper plants positive for ToBRFV [14]. Either the reported symptomatic plants were not harboring the resistance *L* gene [12,13,15], or the symptoms of plants harboring resistance alleles were associated with high temperatures above 30 °C [14]. Specifically, in pepper plants, unlike *Tm-2*^2^ in tomatoes, the defense response NB-LRR encoding *L* gene corresponds with the tobamovirus *Avr* gene encoding the CP for HR activation. The *L* gene of pepper plants has four alleles, *L*^1^–*L*^4^, that confer increasing resistance toward tobamovirus pathotypes P_0_–P_1,2,3,4_. The pathotypes were defined either by infecting *L*^0^ plants only or by the *L* allele the virus overcomes [16]. Regarding the high-temperature response of tobamovirus-infected pepper plants, an *L*^1*a*^ allele showing temperature- and dosage-dependent resistance has been identified. *L*^1*a*^ homozygote plants are susceptible to tobamovirus infections at high environmental temperatures [17,18].

We and others have previously described the HR response of pepper plants harboring *L*^1^, *L*^3^, and *L*^4^ resistance alleles upon ToBRFV inoculations [9,19]. In the face of recent reports on ToBRFV in pepper plants, the possibility that ToBRFV could circumvent *L* gene resistance has led us to study the possible involvement of ToBRFV in symptom development of pepper plants harboring various *L* resistance alleles. We subjected *L*^0^ pepper plants and undefined varieties (UDs), as well as pepper plants harboring *L*^1^*, L*^3^, and *L*^4^ resistance alleles, to ToBRFV mechanical foliar- or truncated-root inoculations. We have previously shown that injured roots of *Solanaceae* plants augmented ToBRFV infection from contaminated soil [20]. Because different defense regulatory pathways are activated in roots and plant leaves [21,22], we carried out both foliar and root inoculations. In our current study, we monitored ToBRFV-inoculated pepper plants for 6–12 months, inspecting any associated symptom manifestations, as well as analyzing ToBRFV infection of leaves, fruits, and seeds. We have found a transient ToBRFV leaf and/or fruit infection in several *L*^1^-, *L*^3^-, and *L*^4^-resistant plants. Infected seeds found in two *L*^4^-resistant plants were noninfectious in a bioassay. One cultivar not harboring the *L* resistance gene was stably infected with the virus. Importantly, fruits of all tested cultivars were asymptomatic.

## 2. Results

### 2.1. Susceptibility of Multiple Pepper Plant Varieties to ToBRFV Infection Was Not Tightly Associated with Disease Manifestations

**Figure 1:**
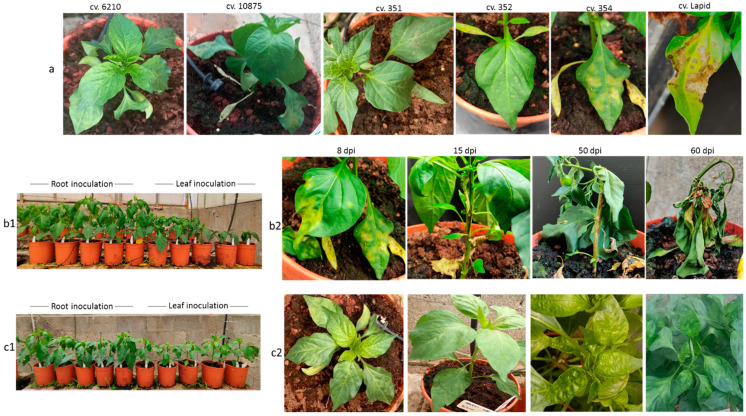
Response of UD pepper plant varieties as well as *L*^0^ and *L*^1^ plants to ToBRFV foliar inoculations: (**a**) *L*^0^ cv. 6210 developed symptoms of yellowing and mosaic; UD plants and *L*^1^ cv. Lapid developed a hypersensitive-like cell death response and HRs, respectively; (**b1**) root- and leaf-inoculated plants cv. 354 at 14 days post-inoculation (dpi); (**b2**) progression of foliar-inoculated cv. 354 toward plant death manifested at 8 dpi, 15 dpi, 50 dpi, and 60 dpi with ToBRFV-infected leaves and stems; (**c1**) root- and leaf-inoculated *L*^0^ cv. 6210 plants at 14 dpi; (**c2**) progression of ToBRFV systemic infection and symptom development in foliar-inoculated *L*^0^ cv. 6210 plants at 8 dpi, 15 dpi, 50 dpi, and 60 dpi.

*L*^0^ cv. 6210, *L*^1^ Lapid, and an additional eight undefined pepper plant varieties (UDs) were subjected to mechanical foliar or root inoculations with ToBRFV (Figure 1). We have recently found that only ToBRFV and not PepMV-IL systemically infects N. tabacum cv. Samsun plants. In order to ensure that the tomato plants were singly infected with ToBRFV and were not coinfected with PepMV-IL, we inoculated N. tabacum cv. Samsun with an inoculum from tomato plants. The systemically infected N. tabacum cv. Samsun plants served for inoculation of tomato plants cv. Ikram, which served as a source for ToBRFV inoculum. These inoculations simulated leaf and soil infections commonly occurring in growing areas. The experiment was conducted during the summertime, with average low and high temperatures of 22.3 °C ± 2.3 °C–31.7 °C ± 2.3 °C. Hypersensitive-like cell death was observed in four UD cultivars, and HRs were observed in L^1^ Lapid at the inoculation sites, which were on both the cotyledons and the first two true leaves beneath the meristem (Figure 1a). Yellowing and shedding of the inoculated leaves followed the HR response. No apparent cell death responses were observed in root-inoculated plants.

**Figure 2:**
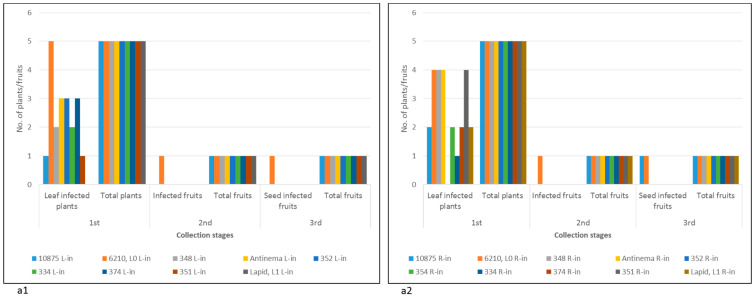
ELISA results of ToBRFV in leaves, fruits, and seeds of UD varieties, *L*^1^ Lapid, and *L*^0^ cv. 6210 plants at several collection stages: (**a1**) ToBRFV in leaf-inoculated UD varieties, *L*^1^ Lapid, and *L*^0^ cv. 6210 plants; (**a2**) ToBRFV in root-inoculated UD varieties, *L*^1^ Lapid, and *L*^0^ cv. 6210 and plants; L, leaf-inoculated; R, root-inoculated.

The UD pepper plant response by cell death, observed at this stage, could be indicative of yet undefined *L* alleles. Alternatively, other mechanisms associated with plant response to pathogens, such as a surge in reactive oxygen species production and nitric oxide, could have initiated cell death response to ToBRFV at the inoculation sites [2]. Severe cell death response manifested in systemic necrosis occurred in one pepper plant, cv. 354, which developed necrotic stems and collapsed at ca. 50 days following foliar ToBRFV inoculations (Figure 1b). Inhibition of growth was apparent at the early stage of 14 days post-inoculation (dpi) (Figure 1(b1)). Plant leaves and stems were infected with ToBRFV, as shown by the enzyme-linked immunosorbent assay (ELISA). Unlike foliar inoculations of cv. 354, root inoculations did not cause similar severe symptoms in that the cultivar and the plants reached the fruiting stage. At 19 dpi, all UD varieties as well as *L*^0^ cv. 6210 and *L*^1^ Lapid plants had ToBRFV-infected leaves. Apparently, the tested plants were susceptible to ToBRFV infection (Figure 2(a1,a2), first collection stage).

**Figure 3:**
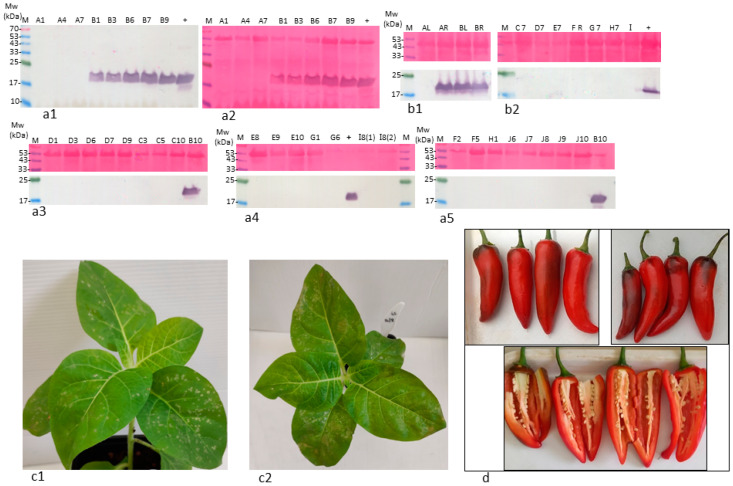
ToBRFV transiently infected leaves of UD varieties and *L*^1^ Lapid plants but stably infected *L*^0^ cv. 6210 plants. (**a1**–**a5**) Western blot analyses (and ponceau-s staining) showed that excluding *L*^0^ cv. 6210 plants, at 75 dpi, leaves of all UD varieties and *L*^1^ Lapid plants were not ToBRFV infected. (**b1**,**b2**) Western blot analyses (and ponceau-s staining) confirmed ToBRFV seed infection only in fruits of *L*^0^ cv. 6210 plants (BL, BR) and in root-inoculated cv. 10875 (AR). (**c1**,**c2**) Necrotic local lesions developed on *N. tabacum* cv. Xanthi inoculated with fruit or seed extract of ToBRFV-infected *L*^0^ cv. 6210 plants (leaf-inoculated). (**d**) Fruits of *L*^0^ cv. 6210 plants were asymptomatic ca. 12 months postinoculation. A–J, all plants of UD varieties, *L*^1^ Lapid (J) and *L*^0^ cv. 6210 (B) were ToBRFV leaf infected at 19 dpi. Numbers indicate the specific plant between 1 and 10 (see Materials and Methods): A, cv. 10875; B, *L*^0^ cv. 6210 plants; L, leaf-inoculated; R, root-inoculated; M, molecular size marker; (+) positive control from tomato plants.

At 75 dpi, leaves were no longer infected by ToBRFV in all tested plants, excluding *L*^0^ cv. 6210, as indicated by sensitive Western blot analyses (Figure 3). Among the tested plants, only *L*^0^ cv. 6210 had ToBRFV-infected fruits at that stage (Figure 2, second collection stage). Seeds of *L*^0^ cv. 6210 and of UD root-inoculated cv. 10875 were infected with ToBRFV, shown in a Western blot, although the ELISA test did not detect fruit infection (Figure 2, second and third collection stages; Figure 3(b1)). However, only seeds of *L*^0^ cv. 6210 were positive in a bioassay (Figure 3(c1,c2)). Local lesions were developed on the test plants, inoculated with either fruit pericarp or seeds of *L*^0^ plants cv. 6210, which were foliar- or root-inoculated with ToBRFV. Western blot analyses also confirmed ELISA data on the uninfected seeds of *L*^1^ cv. Lapid and of the UD cultivars, excluding the root-inoculated plant of cv. 10875 (Figure 2(a1,a2), third collection stage; Figure 3(b1,b2)). At 142 dpi, fruits collected during the growth of all UD cultivars were asymptomatic (Figure S1).

These data indicate that multiple pepper plants, including plants that responded to ToBRFV by hypersensitive-like cell death, were susceptible to a transient ToBRFV infection. However, the infection was dissociated from disease symptoms on fruits. Unlike all the above-tested cultivars, the defined *L*^0^ pepper plants cv. 6210 had a ToBRFV-infected fruit pericarp with infected seeds following both foliar and root inoculations, as shown by both ELISA and Western blots (Figure 2(a1,a2), third collection stage; Figure 3a,b). *L*^0^ pepper plants cv. 6210, which consistently showed ToBRFV infection, reached the fruiting stage, and the fruits lacked any disease symptoms up to 12 months following inoculations (Figure 3d and Figure S1).

### 2.2. Pepper Plants Harboring L^1^, L^3^, or L^4^ Resistance Alleles That Manifest HRs Are Susceptible to ToBRFV Systemic Infection but Show Tolerance toward the Disease

**Figure 4:**
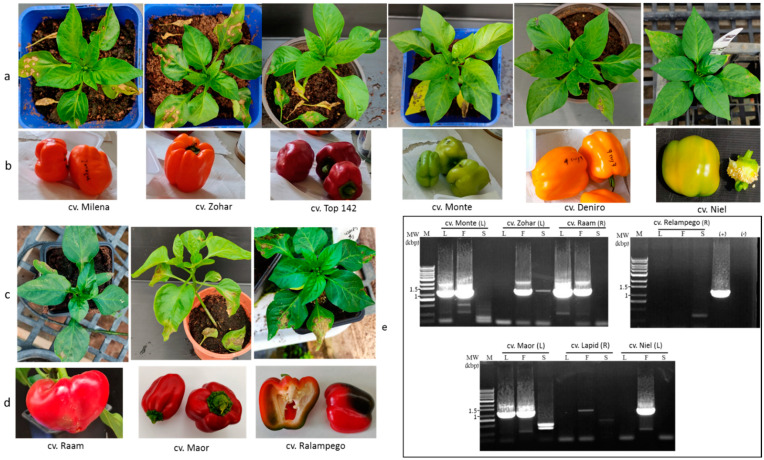
Dissociation between ToBRFV transient infection and disease symptom manifestations on fruits of *L*^1^, *L*^3^, and *L*^4^ pepper plants: (**a**,**c**) leaf-infected plants showed HR manifestations on inoculated leaves; (**b**,**d**) asymptomatic fruits collected from each of the tested cvs.; (**e**) ToBRFV-infected fruits were not seed-infected, excluding *L*^4^ Zohar seeds, as analyzed by double RT-PCR (see Materials and Methods) using primer set number (8); L, leaf; F, fruit; S, seeds; (L), leaf-inoculated; (R), root-inoculated; M, molecular size marker; (+) positive control; (−) negative reaction control.

**Figure 5:**
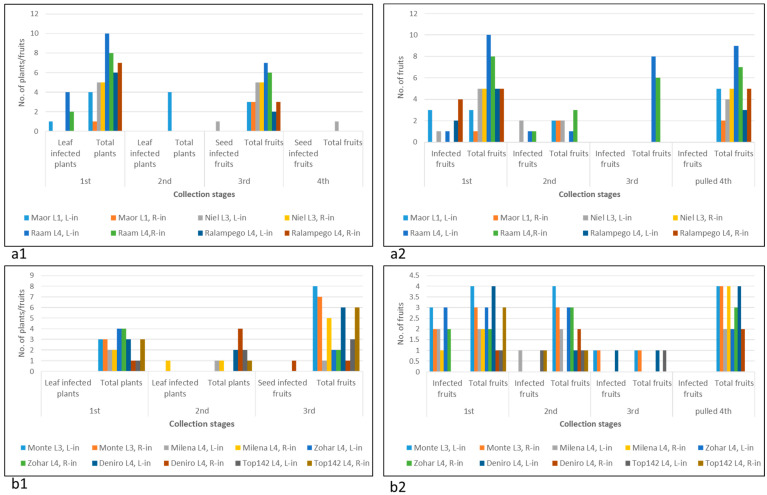
Detection of ToBRFV by ELISA in leaves, fruits, and seeds of leaf- or root-inoculated *L*^1^, *L*^3^, and *L*^4^ pepper cultivars: (**a1**,**b1**) detection of ToBRFV infection of leaves and seeds; (**a2**,**b2**) detection of ToBRFV infection of fruit pericarp; pulled 4th, unscheduled collections of fruits; L, leaf inoculations; R, root inoculations.

The transient ToBRFV infection of *L*^1^ Lapid infers that in pepper plants, ToBRFV pathogenicity changes the effectivity of the NB-LRR HR response [1], preventing complete resistance toward the virus. It seemed possible, therefore, that in pepper plants harboring *L*^1^, *L*^3^, or *L*^4^ resistance alleles that have shown HRs [9], the virus could have systemically infected the plants. We have therefore studied pepper plant growth for ca. 6 months, monitoring ToBRFV leaf and fruit infections in foliar- or root-inoculated *L*^1^, *L*^3^, and *L*^4^ pepper plants. The experiments were conducted during winter and spring seasons, with average low and high temperatures of 9.5 °C ± 1.4 °C–21 °C ± 3.9 °C and 14.9 °C ± 5.1 °C–24.4 °C ± 3.5 °C, respectively. HRs were observed upon ToBRFV foliar inoculations of all tested *L*^1^, *L*^3^, and *L*^4^ cultivars (Figure 4a,c), and inoculated leaf yellowing and shedding followed the HRs. However, in cv. *L*^1^ Maor, *L*^4^ Raam, and *L*^4^ Milena, leaf infection was detected by ELISA test at ~140 dpi (Figure 5(a1,b1), first and second collection stages).

Fruits collected at early pickup stages from several pepper plants harboring the *L* gene were ToBRFV positive (Figure 5(a2,b2)). ToBRFV ELISA-positive fruits were found at either 105–214 dpi (Figure 5(a2), first and second collection stages) or 61–108 dpi (Figure 5(b2), first, second, and third collection stages). Several cultivars had a high percentage of infected fruits such as *L*^1^ Maor, *L*^3^ Monte, *L*^4^ Zohar, and *L*^4^ Ralampego, and two cultivars, *L*^3^ Niel and *L*^4^ Raam, had infected fruits at a late dpi stage. Those cultivars and *L*^1^ Lapid were tested by double RT-PCR (see Materials and Methods) for seed infection (Figure 4e). Although leaves and fruits were ToBRFV-infected, RT-PCR data showed that excluding seeds of an infected fruit of a foliar-inoculated *L*^4^ Zohar plant, seeds of infected fruits of foliar-inoculated cultivars *L*^1^ Maor, *L*^1^ Lapid, *L*^3^ Niel, and *L*^3^ Monte and of root-inoculated cv. *L*^4^ Raam were not infected with ToBRFV (Figure 4e). Importantly, seeds of the two cultivars that were ToBRFV positive by using either the ELISA test (*L*^4^ Deniro) or RT-PCR (*L*^4^ Zohar) were negative in a bioassay performed on *N. tabacum* cv. Xanthi plants. These results indicate the detection of noninfectious virus in the seeds showing CP subunits of noncomplete virions or fragmented RNA. Fruit pericarps of all analyzed *L*^1^-, *L*^3^-, and *L*^4^-resistant cultivars, which were ELISA-positive at early collection stages, were ELISA-negative for ToBRFV at later fruit collection stages (Figure 5(a2,b2), pulled fruits). In addition, fruits of all tested cultivars harboring *L*^1^, *L*^3^, or *L*^4^ resistance alleles during all collection stages were asymptomatic, indicating the dissociation between ToBRFV transient infections and disease symptom manifestations on the fruits (Figure 4b,d).

### 2.3. ToBRFV in Stably Infected L^0^ Pepper Plants cv. 6210 Was Infectious

**Figure 6:**
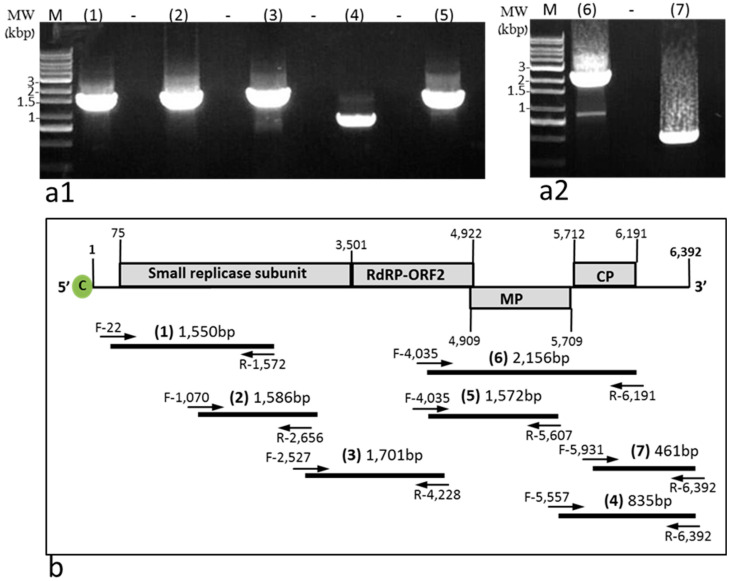
Infectious ToBRFV in systemically infected *L*^0^ cv. 6210 plants: (**a1**,**a2**) RT-PCR of ToBRFV in leaves of systemically infected *L*^0^ cv. 6210 plants using the primer sets 1–7 illustrated in (**b**); (**b**) a scheme of ToBRFV genome showing total genome coverage by the amplicons amplified in (**a1**,**a2**).

ToBRFV systemic infection of *L*^0^ pepper plants cv. 6210 was stable, and the virus was infectious, as tested by a biological assay on *N. tabacum* cv. Xanthi plants (Figure 3(c1,c2)). ToBRFV-infected leaves of *L*^0^ plants cv. 6210 were analyzed by RT-PCR for the presence of ToBRFV using amplifications of seven segments covering the whole genome of the virus (Figure 6(a1,a2),b). These data further support the infectious potential of ToBRFV in the stably infected *L*^0^ cv. 6210 plants. At the fruit harvest stage (4 months postinoculation), the genome sequence of ToBRFV in *L*^0^ cv. 6210 plants was resequenced, and the obtained sequence was highly similar to the original ToBRFV sequence (GenBank accession no. KX619418) from *Tm-2*^2^-resistant tomato plants. We have identified four nucleotide changes at positions: G1,310A, T2,399A, C2,401T, and A2,531T. Only C2,401T was nonsynonymous, causing the substitution of alanine for valine in the small replicase subunit. This sequence similarity infers that no sequence adaption in the movement protein and the coat protein was required for ToBRFV systemic infection in pepper plants.

**Figure 7:**
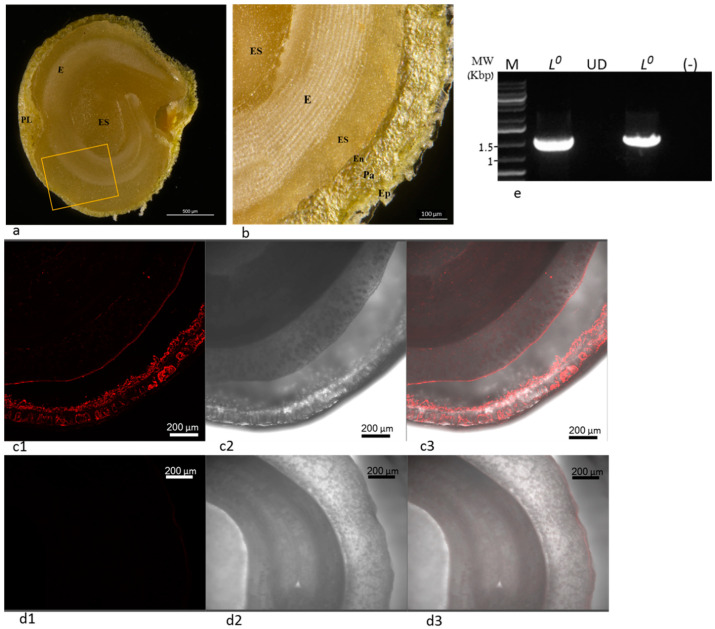
ToBRFV in seeds of systemically infected *L*^0^ cv. 6210 plants were not restricted to epidermis surface but localized in the maternal parenchyma and endothelium: (**a**) a bright light image of a longitudinal dissected seed; (**b**) higher magnification of the area marked by a box in (**a**); (**c1**–**c3**) seeds of ToBRFV foliar-inoculated *L*^0^ cv. 6210 (**d1**–**d3**); seeds of ToBRFV foliar-inoculated *L*^1^ cv. Lapid serving as a negative control for ToBRFV seed infection; (**c1**,**d1**) a red channel showing ToBRFV in the epidermis, parenchyma, and endothelium not entering the endosperm of *L*^0^ cv. 6210 plants and not in *L*^1^ cv. Lapid plants (the same setting used for the two images); (**c2**,**d2**) a transmitted light image; (**c3**,**d3**) merging transmitted and red channel images; (**e**) RT-PCR showing the presence of 1122 ToBRFV amplicon in seeds of leaf- and root-inoculated *L*^0^ cv. 6210 plants using primer set number (5); Ep, epidermis; Pa parenchyma; En, endothelium; ES, endosperm; E, embryo.

In order to confirm the establishment of ToBRFV in the infected *L*^0^ cv. 6210 plant tissues, we have examined the plant seeds for virus distribution using in situ immunofluorescence (Figure 7). ToBRFV localization in the infected seeds, studied by in situ immunofluorescence, revealed that ToBRFV infected the inner maternal-derived tissues (Figure 7(c1–c3)). Apparently, ToBRFV in seeds of foliar-inoculated *L*^0^ cv. 6210 plants were not restricted to the epidermis of the seed coat but were localized in the inner parenchyma and in the endothelial cells bordering the endosperm as well (Figure 7a,b,(c1–c3)). Seeds of *L*^1^ Lapid that were ToBRFV-negative, shown by a Western blot and double RT-PCR (see Materials and Methods) (Figure 3(b2,4e)), served as a negative control (Figure 7(d1–d3)). ToBRFV-infected seeds of *L*^0^ plants cv. 6210 showed a ToBRFV PCR product of 1122 bp (Figure 7e). These data further indicated that foliar-inoculated *L*^0^ cv. 6210 plants had a stable systemic infection of ToBRFV, which allowed the virus to penetrate the parenchyma and the endothelium through the maternal precursor tissues [23]. However, ToBRFV seed infection was not associated with disease symptom manifestations on fruits of *L*^0^ cv. 6210 plants (Figure 3d and Figure S1).

## 3. Discussion

We have shown that pepper plants harboring the *L* resistance alleles could have a transient systemic infection of ToBRFV, but the infection was dissociated from disease symptom manifestations on fruits and seeds. ToBRFV detected in seeds of two *L*-resistant cultivars was not infectious. Transient systemic infection occurred, although HRs were observed on the inoculated leaves. It has been previously documented that depending on the pathogen plant, resistance genes could activate HRs, which were not associated with resistance response [1]. HRs could be misleading in terms of plant susceptibility to viral infection, manifested in systemic infection of the pathogen. However, regarding ToBRFV infection of *L*-resistant pepper plants, the HRs were associated with a transient viral systemic infection and tolerance toward the viral disease [24].

HR induction is temperature-dependent, and high temperatures lead to increased susceptibility of the plants to the pathogen. Low temperature-dependent induction of HRs and high temperatures associated with an increase in plant susceptibility to pathogens have been observed in many plant–pathogen interactions, including bacteria fungi or viruses, such as *Pseudomonas*, *Puccinia graminis*, and capsicum chlorosis virus, as well as tobacco mosaic virus, respectively [4,25,26,27,28]. However, in several cases, the high temperatures induced recovery [28]. We conducted our experiments during the winter, spring, and summer seasons, and the plants grown in a greenhouse were exposed to a wide temperature range of 10 °C to 30 °C. Our results, therefore, simulated uncontrolled field conditions occurring in multiple growing areas in the world.

Because we did not carry out our inoculation experiments under controlled high-temperature conditions, it could be that ToBRFV pathogenicity was the causal factor in compromising the HR resistance effect. It has been shown that miRNAs regulate NB-LRR protein expression [29,30,31]. It seems possible, therefore, that the silencing suppressor protein of ToBRFV would affect NB-LRR family members other than tomatoes’ *Tm-2*^2^ via interference with miRNA regulatory pathways. We hypothesize that in pepper plants, deregulation of miRNA regulatory loops by the silencing suppressor of ToBRFV rendered NB-LRR-associated HR less effective in the resistance response toward the pathogen systemic infection, but resistance toward disease manifestations was not impaired, and further studies may shed light on the role of RNA the silencing suppressor. The difference between root and plant leaf regulatory pathways of RNA silencing [21,22] could be the source of differences found in systemic ToBRFV infection efficacy of foliar- and root-inoculated *L*-resistant pepper plants, as well as the systemic necrosis that occurred only in foliar-inoculated cv. 354 plants.

Our finding of the stable systemic infection in *L*^0^ cv. 6210 plants emphasizes the difference between pepper plants harboring the *L* resistance gene and *L*^0^ plants. However, the asymptomatic fruits and seeds indicate that in pepper plants, under conditions of stable systemic ToBRFV infections, there could be a dissociation between systemic infection and disease manifestations. This tolerance toward the disease allowed the preservation of the crops but should alert growers of tomato plants regarding a possible disease inoculum source in the ToBRFV-infected *L*^0^ pepper plants. Putatively, ToBRFV transient systemic infection of pepper plants harboring the *L* resistance alleles could constitute a source of infection for tomato plants.

## 4. Materials and Methods

### 4.1. Tested Plants, ToBRFV Inoculations, and Biological Assays for Infectious Virus

The following pepper plant cultivars were analyzed for ToBRFV systemic infections: one cultivar not harboring the *L* gene cv. 6210 (b); eight cultivars of undefined genotypes, 10,875 (a), 348 (c), Antinema (d), 352 (e), 354 (f), 334 (g), 374 (h), and 351 (i); two cultivars harboring the *L*^1^ resistance allele, Lapid (j) and Maor; two cultivars harboring the *L*^3^ resistance allele, Niel and Monte; and six cultivars harboring the *L*^4^ resistance allele, Raam, Ralampego, Milena, Zohar, Top 142, and Deniro. The multiple pepper cultivars served for confirmation of the results, serving as controls for each other. Each cultivar was either foliar (5–10 plants) or root (5–10 plants) sap-inoculated with ToBRFV via mechanical inoculation using an inoculum source from ToBRFV-infected *Tm-2*^2^-resistant tomato plants cv. Ikram. For foliar inoculations, the cotyledons and the first two true leaves beneath the meristem were inoculated, and for root inoculations, truncated roots were dipped in the inoculum solution.

In recent years, tomato plants in Israel have mostly been coinfected with ToBRFV and the potexvirus Pepino mosaic virus (PepMV-IL) [32]. Therefore, we used systemically infected *N. tabacum* cv. Samsun plants for the inoculation of tomato plants cv. Ikram. Leaves of ToBRFV (GenBank accession no. KX619418)-infected tomato plants cv. Ikram, which were confirmed to contain ToBRFV only by ELISA and Western blot using specific antibodies for ToBRFV and PepMV [32], were crushed in 0.01 M sodium phosphate buffer pH = 7.0 and served for inoculation of the pepper plants.

The ToBRFV-infected and negative control pepper plants (noninoculated plants) were grown in an experimental greenhouse and inspected for symptom manifestations. The upper leaf beneath the meristem was sampled for the various tests of ToBRFV infection. All the fruits were collected and inspected for symptoms and ToBRFV infection for ca. 6 months. The experiments with plants harboring the *L* resistance alleles were conducted during winter and springtime, and the plants were exposed to a wide temperature range, with average low and high temperatures of 9.5 °C ± 1.4 °C–21 °C ± 3.9 °C and 14.9 °C ± 5.1 °C–24.4 °C ± 3.5 °C, respectively. The experiments with ToBRFV-infected UD varieties, *L*^1^ Lapid, and *L*^0^ cv. 6210 plants were conducted during the summertime, with average low and high temperatures of 22.3 °C ± 2.3 °C–31.7 °C ± 2.3 °C (https://ims.data.gov.il/ims/1), accessed at the time range 1 January 2021–31 August 2021. *L*^0^ cv. 6210 plants were left to grow for ca. 12 months.

Biological assays were conducted by inoculating *N. tabacum* cv. Xanthi plants for local lesion manifestations using seed or fruit pericarp extractions in 0.01 M sodium phosphate buffer pH = 7.0.

### 4.2. Indirect Enzyme-Linked Immunosorbent Assay (ELISA)

Leaf samples, as well as fruit pericarp and washed seeds (24 h in water), were subjected to indirect ELISA test, principally as previously described [32]. Samples were ground in coating buffer (Agdia) at a ratio of ~600 µL/µg sample and incubated with 1:5000 dilution of specific antibodies against ToBRFV [9] in PBS for 3 h at 37 °C. For detection, alkaline phosphatase (AP)-conjugated goat antirabbit IgG (Sigma, Steinheim, Germany, 1:5000 in PBS) and p-nitro phenyl phosphate substrate (Sigma, 0.6 mg/mL) were used. Data were recorded at 405 nm and 620 nm, and O.D. values that were 2.5 times the value of the negative controls were considered positive.

### 4.3. Western Blot Analysis

ToBRFV-inoculated pepper plant leaves or seeds were subjected to protein extraction with urea-SDS-β-mercaptoethanol (USB) buffer, principally as previously described [32]. A constant ratio of 5.5 µL/µg tissue sample or 5.5 µL/seed (50 seeds/sample) was kept in all tested samples. The electro-blotted nitrocellulose membranes were subjected to ToBRFV detection using specific antibodies against ToBRFV [9]. AP-conjugated goat antirabbit antibodies (Sigma) were used for detection with NBT and BCIP (Bio-Rad, Hercules, California, USA) substrate. Ponceau-s staining of total loaded proteins was conducted following ToBRFV-CP detection in order to avoid ponceau-s background in CP detection.

### 4.4. Reverse Transcription (RT)-PCR

Fruit pericarp, leaf, and seed samples were ground in general extraction buffer (Bioreba, Reinach, Switzerland) and subjected to viral RNA extraction using Accuprep viral RNA Extraction kit (Bioneer, Daejeon, Korea). Reverse transcription was conducted using qPCRBIO cDNA synthesis kit (PCR Biosystems, London, UK). PCR amplification was performed using eight different primer sets. Primer sets 1–7 were used for the whole ToBRFV genome coverage. (1) For an amplicon size of 1550 bp, F-22 (5′ CCAACAACAACAAACAACAAACA 3′) and R-1572 (5′ CTAATGCGTCTCCCGACACT 3′). (2) For an amplicon size of 1586 bp, F-1070 (5′ TTACAGCGCAATGGAAGATG 3′) and R-2656 (5′ GCCTGCTTACCCGGTACTAA 3′). (3) For an amplicon size of 1701 bp, F-2527 (5′ ATGGAGAGCCTCATGTCAGC 3′) and R-4228 (5′ AGCTGGCGTCTTCCTTGTAA 3′). (4) For an amplicon size of 835 bp, F-5557 (5′ TTTAGTAGTAAAAGTGAGAAT 3′) and R-6392 (5′ TGGGCCCCTACCGGGGGT 3′). (5) For an amplicon size of 1572 bp, F-4035 (5′ GGCCTTGCAGACGATTGTGTA 3′) and R-5607 (5′ TGCAAGCCTTACAGACATCG 3′). (6) For an amplicon size of 2156 bp, F-4035 (5′ GGCCTTGCAGACGATTGTGTA 3′) and R-6191 (5′ TCAAGATGCAGGTGCAGAG 3′). (7) For an amplicon size of 461 bp, F-5931 (5′ ACAATGCGGTACTAGATCCTCT3′) and R-6392 (5′ TGGGCCCCTACCGGGGGT 3′). (8) For an amplicon size of 1122 bp, F-1534 (5′ AGATTTCCCTGGCTTTTGGA 3′) and R-2656 (5′ GCCTGCTTACCCGGTACTAA 3′). Amplicons were Sanger sequenced (Hylabs, Rechovot, Israel). In selected samples from plants harboring the *L* alleles that showed no visible amplicons, the PCR was repeated twice on 1 µL of the first PCR amplification mixture, serving as a template for the second PCR amplification using the same primers (double RT-PCR).

### 4.5. In Situ Immunofluorescence

Seeds of *L*^0^ pepper plants cv. 6210, which were ToBRFV foliar-inoculated, as well as seeds of *L*^1^ Lapid-inoculated plants, were washed in water for 24 h before being subjected to longitudinal dissection and in situ immunofluorescence using specific antibodies against ToBRFV. The procedure was principally conducted as described before [32], with few modifications. Samples were fixed for 2 h at a room temperature with a fixation buffer containing formaldehyde (4%, *v*/*v*) and glutaraldehyde (0.2%, *v*/*v*) in 50 mM PIPES and 1 mM CaCl2, pH = 7.0. After washing twice with PBS-Tween-20 (0.05%, *v*/*v*), samples were blocked with PBS-skim milk (1%, *w*/*v*) for 30 min and then incubated with specific antibodies against ToBRFV [9], using a 1:4000 dilution factor in PBS-skim milk, overnight at 4 °C. Samples were washed twice with PBS-Tween and incubated for 1.5 h at 37 °C with goat antirabbit IgG conjugated to Alexa Fluor 594 (Invitrogen, Carlsbad, CA, USA) at 1:1000 dilution in PBS with agitation at 100 rpm. Image acquisition was performed using a Leica SP8 laser scanning microscope (Leica, Wetzlar, Germany) equipped with solid-state lasers with 552 nm light, HC PL APO CS 10x/0.40 objective (Leica, Wetzlar, Germany), and Leica Application Suite X software (LASX, Leica, Wetzlar, Germany). Red emission signals were detected with PMT detector in the range of 560–650 nm.

## 5. Conclusions

We have shown that *L* resistance gene was not broken by ToBRFV single inoculation of pepper plants harboring *L*^1^, *L*^3^, or *L*^4^ alleles. However, HRs or hypersensitive-like cell death responses showed compromised resistance toward the virus.

- Both foliar- and root-inoculated plants harboring the *L* alleles were susceptible to a transient ToBRFV infection, which was not associated with symptomatic fruits.
- A severe cell death response and wilting occurred in one foliar-inoculated undefined cultivar (cv. 354) that showed systemic necrosis and did not reach the fruiting stage. The root-inoculated plants of this cultivar had asymptomatic fruits.
- A stable systemic ToBRFV infection occurred in defined *L*^0^ pepper plants, in which the virus was localized to the maternal origin of the seed tissues (epidermis, parenchyma, and endothelium). ToBRFV in the *L*^0^ plants was infectious and highly similar to the original ToBRFV sequence from tomato plants, indicating low constraints were imposed on ToBRFV by the *L*^0^ pepper host. Similar to *L* gene-resistant plants, there was a dissociation between ToBRFV systemic infection of the *L*^0^ plants and disease symptoms on fruits.

Regarding a disease management conclusion, susceptible *L*^0^ pepper plants could constitute a primary source of infection and mediate disease spread when grown in proximity to tomato plants.

## Data Availability

Not applicable.

## Supplementary Materials

The following supporting information can be downloaded at: https://www.mdpi.com/article/10.3390/plants11182378/s1. Figure S1, Dissociation between transient ToBRFV leaf infection and disease symptoms on fruits.
