# Supplementary material for: Pepper Plants Harboring L Resistance Alleles Showed Tolerance toward Manifestations of Tomato Brown Rugose Fruit Virus Disease
Source: Plants (Basel). 2022 Sep 12;11(18):2378. doi: 10.3390/plants11182378 (PMC9506004; doi:10.3390/plants11182378)
Supplement: Supplementary file 1 [file plants-11-02378-s001.zip › plants-1903367-supplementary.pdf]

Supllementary file

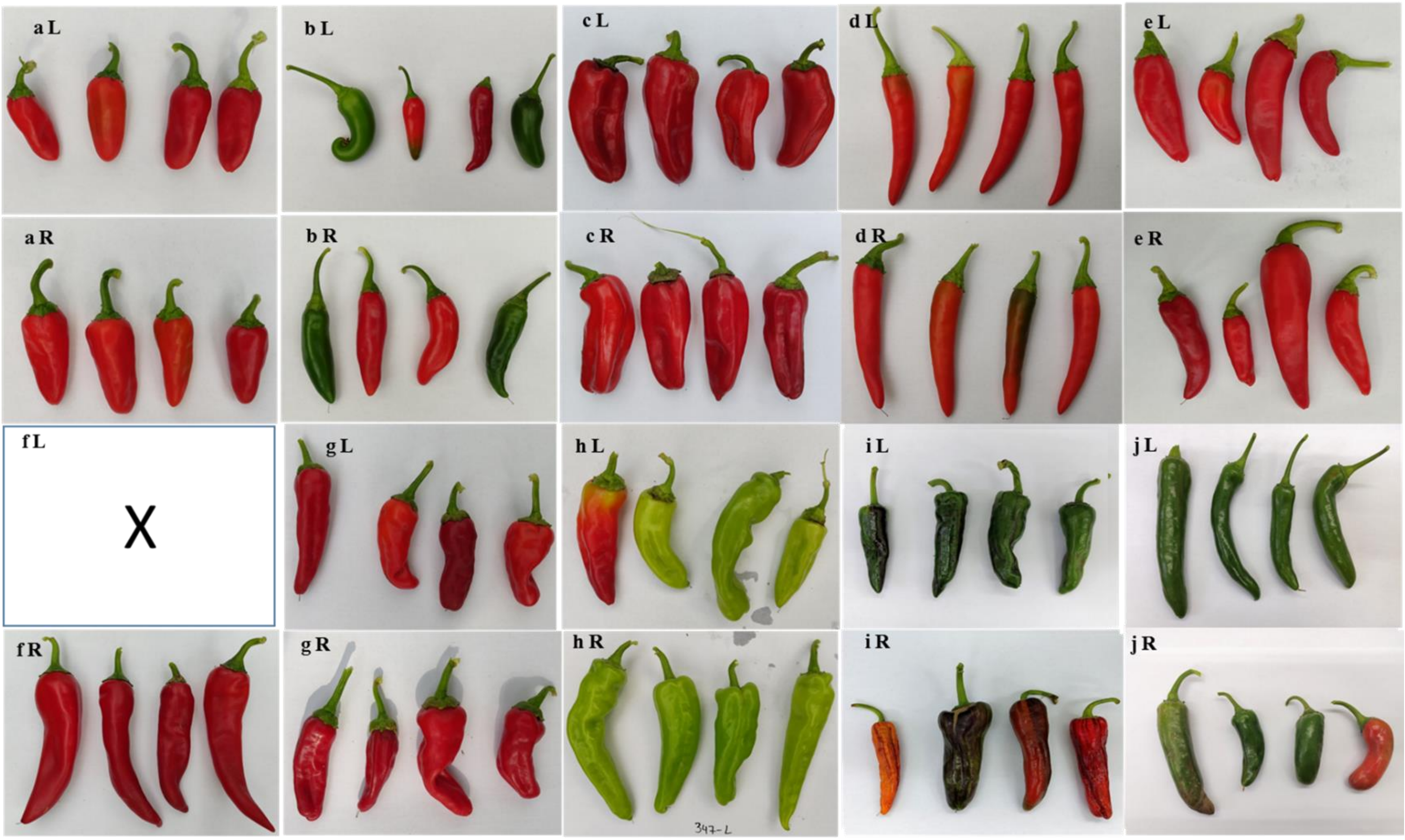

Figure S1. Dissociation between transient ToBRFV leaf infection and disease symptoms on fruits. Fruits were collected at 142 dpi from L0 cv. 6210 plants (b), 8 UD plants (a, c-i) and L1 cv. Lapid (j) were Asymptomatic. Leaf-inoculated cv. 354 plants collapsed and did not reach the fruiting stage (f). L, leaf-inoculated; R, root-inoculated.
